# Immune priming of honey bees protects against a major microsporidian pathogen

**DOI:** 10.1002/ps.70106

**Published:** 2025-08-04

**Authors:** James C. Nieh, Matthew Endler, Andrey Rubanov, Alexander Neskovic, Xianbing Xie, Honghong Li, Zachary Y. Huang

**Affiliations:** ^1^ School of Biological Sciences, Department of Ecology, Behavior, and Evolution University of California San Diego La Jolla CA USA; ^2^ Department of Entomology Michigan State University East Lansing MI USA; ^3^ Department of Laboratory Animal Science Nanchang University Nanchang China; ^4^ Institute of Pesticide and Environmental Toxicology Guangxi University Nanning China

**Keywords:** honey bee health, *Nosema ceranae*, *Apis mellifera*, infection, pathogen, Toll pathway

## Abstract

**BACKGROUND:**

Honey bees face significant threats from pathogens like *Nosema ceranae*, a microsporidian parasite that contributes to global colony declines. Immune priming, exposure to pathogen antigens to stimulate protective responses, could mitigate infection risks. We tested whether priming honey bee larvae and adults with heat‐killed *N. ceranae* spores reduces susceptibility to subsequent live infections in laboratory and field conditions.

**RESULTS:**

Priming consistently lowered infection levels and rates. The strongest effect occurred in laboratory‐reared larvae, with a 97% reduction in mean spore counts relative to unprimed controls. Newly emerged adults primed and maintained in field colonies showed a 56% decrease, larvae primed by nurse bees in colonies had a 52% reduction, and newly emerged adults primed in laboratory cages showed a 34% reduction. Primed adults in field colonies also exhibited increased survival following live pathogen exposure as compared to positive controls. However, immune priming alone sometimes reduced bee lifespan. Priming altered expression of Toll pathway immune genes associated with *Nosema* resistance, particularly defensin‐1.

**CONCLUSION:**

Immune priming significantly protects honey bees from *N. ceranae* infection, reducing pathogen loads and enhancing survival across diverse conditions. Despite potential longevity trade‐offs, these findings support immune priming as a viable strategy to improve honey bee health and to reduce microsporidian‐induced colony losses. © 2025 The Author(s). *Pest Management Science* published by John Wiley & Sons Ltd on behalf of Society of Chemical Industry.

## Graphical abstract

We immune‐primed honey bees at two developmental stages in the laboratory and the field with heat‐killed *Nosema ceranae* spores. When subsequently fed live spores, immune‐primed adults had lower infection levels.

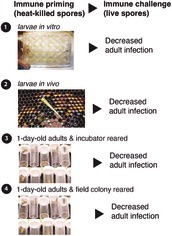

## 1 INTRODUCTION

Insect immune priming occurs when prior exposure to a sublethal dose of a pathogen or pathogen‐derived material elevates an insect's immune response, thereby improving defense against future infections.^1^, ^2^, ^3^ This priming can be highly specific, targeting a particular pathogen, or more general, activating broader immune pathways.^4^ Importantly, such priming can operate on both short‐ and long‐term timescales and, in some cases, can even extend across generations through trans‐generational immune priming. For example, Dickel *et al*.^5^ and Hernández López *et al*.^6^ showed that honey bee queens fed inactivated *Paenibacillus larvae* bacilli produced offspring that had enhanced survival upon exposure to this same bacterial pathogen.

Although the mechanisms underlying insect immune priming are not fully understood, recent findings have shed light on how insects may retain a form of ‘immune memory.’ Within an individual, transstadial immune priming occurs when the immune system is primed during one developmental stage and this immunity is passed to the next stage.^7^ In transgenerational immune priming, lipophorins and vitellogenins can transfer microbes or microbe fragments between insect mothers and their offspring.^8^ For example, Salmela *et al*.^9^ showed that the egg‐yolk protein vitellogenin can bind pathogen cell wall components and transport them into offspring to elicit trans‐generational immune priming. In addition epigenetic modifications such as DNA methylation, histone acetylation, microRNAs, and pathogen recognition receptors can play roles in maternal and paternal transgenerational immune priming.^7^, ^8^ In bumblebees, Barribeau *et al*.^10^ demonstrated that trans‐generational immune priming induced by injecting queens with heat‐killed bacteria can alter offspring gene expression to mimic a primary immune response, and similar patterns have been observed in honey bees, including viral (deformed wing virus) trans‐generational immune priming.^11^ Complementing these genetic and epigenetic insights, Lopez *et al*.^6^ reported that immune priming of honey bees with heat‐killed *P. larvae* stimulated the differentiation of prohaemocytes into haemocytes.

In honey bees (*Apis mellifera*), immune priming involves a variety of inducible defenses at both cellular and molecular levels, notably the production of anti‐microbial peptides such as abaecin, defensin‐1, defensin‐2, hymenoptacin, apidaecin, and apisimin.^9^, ^12^, ^13^, ^14^, ^15^ Similar to *Drosophila*, which initiates Toll‐pathway activation when primed by heat‐killed bacteria,^16^ honey bees use Toll‐mediated immune responses to target pathogen metabolism and membrane integrity. This Toll‐centric defense is especially relevant for combating *Nosema*, a genus of fungal‐related intracellular parasites that can severely compromise honey bee health and colony survival.^17^ One species in particular, *Nosema ceranae*, has gained attention due to its global distribution and its synergistic impacts on honey bees when combined with other stressors.^18^, ^19^ Although a taxonomic reclassification to *Vairimorpha ceranae* was proposed,^20^ we follow the more recent consensus of Bartolome *et al*.^21^ in using its original genus name. *Nosema ceranae* primarily infects midgut epithelial cells^22^, leading to tissue rupture,^23^ malnutrition,^24^ and reduced longevity.^25^ Current treatment hinges on the antibiotic fumagillin, which may be losing efficacy^26^ and is only effective against the vegetative stage of *Nosema* infection.^5^ Moreover, this antibiotic is banned for use by beekeepers in the European Union.^27^

Thus, given the issues confronting the use of the most effective treatment against *N. ceranae*, we decided to test if immune priming could protect worker bees from this common and globally distributed pathogen. Multiple studies have demonstrated the efficacy of using heat‐killed pathogens to immune prime bumble bees.^28^, ^29^, ^30^ Dickel *et al*.^5^ used inactivated *P. larvae* vegetative stage bacilli to immune prime honey bee queens. Lopez *et al*.^6^ immune primed honey bee queens with small doses of live *P. larvae* bacilli, but we decided to use heat‐killed spores because Eiri *et al*.^31^ observed that feeding honey bee larvae heat‐killed *N. ceranae* spores did not cause infection, raising the possibility of using these spores as safe immunostimulants. Innate immune strength in *Apis mellifera* varies across developmental stages, and larvae and pupae typically have higher hemocyte counts than adults.^32^ We chose larvae and adults for our immune priming treatment because both stages actively feed, making oral administration of heat‐killed spores feasible. Honey bees cease feeding at the end of the larval period and resume only as adults.^33^ Additionally, adult bees were included because previous studies have successfully demonstrated immune priming in adult queens.^5^, ^6^ We therefore tested whether feeding honey bee larvae or newly emerged workers heat‐killed *Nosema* spores can induce immune priming and confer protection against this disease.

Research on *Apis mellifera* immune defenses against *N. ceranae* infection has focused on the activation of five Toll AMP (antimicrobial peptide) genes (*abaecin*, *hymenoptacin*, *defensin‐1*, *apidaecin*, and *defensin‐2*) that are up‐regulated during immune defense against *N. ceranae*.^34^ Huang *et al*.^35^ showed that honey bees selected for tolerance to *N. ceranae* likewise up‐regulate multiple Toll pathway immune genes, including all six AMP genes. We therefore hypothesized that primed bees would have lower infection levels than non‐primed controls and display up‐regulation of Toll‐pathway AMPs.

## 2 MATERIALS AND METHODS

### 2.1 Study site and colonies

We conducted four experiments, testing the effects of immune priming on larvae grown *in vitro* (experiment 1, E1), larvae fed by nurse bees inside full‐sized colonies (*in vivo*, experiment 2, E2), 1‐day‐old adults emerging from combs and subsequently reared in incubated cages (experiment 3, E3), and 1‐day‐old adults that were then reared in field colonies (experiment 4, E4). Experiments E1–E3 were conducted at University of California San Diego (UCSD), La Jolla, CA, USA over 3 years (one experiment per year) with Koehnen & Sons, Inc. package *Apis mellifera ligustica* freely mated queens, and E4 was conducted at Michigan State University (MSU), East Lansing, MI, USA over one summer (these *Apis mellifera ligustica* colonies containing a mix of predominantly European subspecies, with freely mated queens). In total, we used 30 different colonies (eight colonies E1, ten colonies E2, nine colonies E3, and three colonies E4). For all experiments, we obtained new package colonies in the Spring and eliminated potential *Nosema* infections by feeding all colonies Fumagilin‐B in 25 mg/L of 2.0 m sucrose solution (3.8 L/colony). After this initial treatment, we did not use the colonies for 60 days to allow the antibiotic to dissipate (method of Milbrath *et al*.^25^) and to reduce the potential remaining effects of Fumagilin‐B on workers that we tested. Although fumagilin can alter honey bee midgut proteins at 10 days after treatment, the duration of these effects is not clear.^36^ However, this initial treatment was applied uniformly across all colonies, ensuring that all experimental groups experienced the same baseline exposure. As such, any residual influence of fumagillin would have been consistent across treatments.

Data from Milbrath *et al*.^25^ and this article show that bees can become highly infected with *N. ceranae* after these 60 days of antibiotic dissipation, allowing us to have infected bees as positive controls. During our trials, we followed standard methods to assess individual and colony‐level infection levels.^37^ To assess colony‐level infection, we haphazardly captured 20 foragers (bees returning with pollen) per colony at colony entrances and used hemocytometer counts of their dissected midguts (see method later) to verify that the colonies were not infected with *Nosema* (standard methods, see Fries *et al*.^37^).

### 2.2 Maintaining and harvesting *N. ceranae* stock

*Nosema ceranae* spores were obtained from heavily infected *Apis mellifera ligustica* workers from the Nieh laboratory apiary in La Jolla, CA, USA. Spore species were verified with polymerase chain reaction (PCR) (see later). To generate spore stock, we placed 25 newly emerged worker bees into a cage equipped with a syringe containing 5 mL of 2.0 m sucrose solution mixed with one million *N. ceranae* spores (40 000 spores per bee). Bees were then fed pure 2.0 m sucrose (with no spores) *ad libitum* and given 10–12 days to develop heavy infections,^37^ after which the midguts of these bees were dissected out. We followed this same procedure for all experiments over time, generating fresh spore stock from the same original spore source. Three midguts from infected bees were placed in 100 μL double‐distilled water (ddH_2_O) in a 1.5 mL Eppendorf tube, ground with Kimble polypropylene pestles, diluted to 1 mL, vacuum filtered through a Buchner funnel lined with Fisherbrand P8 filter paper, and concentrated by centrifuging for 15 min at 6702 × *g* for 6 min (Eppendorf 5415D Centrifuge, Hamburg, Germany). The supernatant was discarded and the precipitates were combined and re‐suspended in 500 μL of ddH_2_O (modified from Webster *et al*.^38^). A hemocytometer with the re‐suspended solution was observed at 400× total magnification under a bright‐field light microscope (Zeiss Axioskop, Oberkochen, Germany) following the methods of Cantwell^39^ to determine spore concentrations. In our protocol, a single spore corresponded to 5000 midgut spores, thus this number represents the technique's margin of error. Spores were harvested within 12 h of each new trial and stored at 4 °C before use in the trial to ensure viability.^40^

Heat‐killed spore solutions were prepared by autoclaving spores for 30 min at 121 °C.^41^ Each sterilized sample was subsequently recounted and its volume was adjusted with sterile ddH_2_O to ensure the correct *Nosema* concentration because autoclaving slightly reduced the solution volume. Autoclaved spores were viewed with a Zeiss Axioskop microscope and found to be fully intact. We stored these spores at −20 °C in multiple 1.5 mL Eppendorf tubes. When needed, we thawed out the heat‐killed spore solution for 24 h at 4 °C and then brought it to room temperature before feeding it to the bees. Autoclaving killed all the spores because no bees that were fed autoclaved spores subsequently developed infections,^31^ see also later.

For E4, we mailed freshly prepared live spores (propagated from the original spore stock) that were frozen at −70 °C, along with autoclaved spores, all packed with dry ice, to MSU. Frozen spores have lower viability than fresh spores, and thus, the Huang laboratory fed these frozen spores to workers to generate fresh spore stock as described earlier. Spores used in all experiments, therefore, had a common origin.

DNA was extracted to confirm we were using *N. ceranae*. As earlier, we centrifuged dissected honey bee midguts with an estimated 4 × 10^4^ spores per microliter to form a concentrated pellet that we froze with liquid nitrogen and then crushed with a pestle before DNA extraction with a Bioneer Accuprep Genomic DNA extraction kit. We used primer pairs NoscRNAPol‐F2 (5′‐TGGGTTCCCTAAACCTGGTGGTTT‐3′) and NoscRNAPol‐R2 (5′‐TCACATGACCTGGTGCTCCTTCT‐3′).^42^ The resulting spore DNA was then amplified with PCR and sequenced using standard methods.^31^ We then checked Genbank sequences and confirmed that our spores were *N. ceranae*. We conducted this confirmation for all four experiments.

For all other experiments, we hemocytometer‐counted *Nosema* spores instead of using quantitative PCR (qPCR) to measure *Nosema* infections. Although qPCR is highly sensitive and can be used to measure the level of infection,^43^ it does not distinguish between the different life stages of *Nosema*. We were focused on the infectious spore stage since we wished to test if immune priming can reduce the propagation of *Nosema* in a colony. We also wished to compare our data on *Nosema* infection with other studies that have correlated spore counts with immune gene activation.^35^, ^44^ Spore counts and gene expression were measured in separate individuals because high‐quality RNA required immediate flash‐freezing upon collection, which was not possible for bees used in survival assays. Bees for spore counts were collected at death, while gene expression required live sampling to preserve RNA integrity.

### 2.3 Treatments

In all experiments, we had four groups that arose from the combination of primary and secondary exposure treatment groups. The primary treatment was control or immune priming followed by a secondary treatment (control or pathogenic live spores) in sugar solutions. Specifically, in the primary treatment, bees were fed no *Nosema* spores (0) or 40 000 heat‐killed spores (IP). In the secondary treatment, they were fed no *Nosema* spores (0) or 40 000 (40) freshly harvested *Nosema* spores. This yielded four different groups: 0‐0 (negative control, no spores ever fed to bees), 0‐40 (positive control, bees received no immune priming treatment and then fed live spores), IP‐0 (immune priming control, immune primed bees receiving no live spores), and IP‐40 (immune primed bees subsequently fed live spores). For simplicity, we will refer to these four groups as four treatments.

### 2.4 Measuring immune gene activation

For all four experiments, we measured the activation of four AMP immune genes in the Toll pathway, *abaecin*, *defensin‐1*, *apidaecin*, and *hymenoptacin* (primers in Supporting Information, Table S2), that were shown to be up‐regulated in bees infected by *N. ceranae* ^45^ and two reference genes, *actin* ^45^ and *GAPDH*.^46^ For our first experiment, E1, we conducted limited analyses, which we expanded for all subsequent experiments, given our finding of significant gene activation in E1 (see Results section). Sample sizes are shown in Table S1, and primers are listed in Table S2. At the specific age (see details later), we flash‐froze each living bee in liquid nitrogen, placed its abdomen in RNAlater, and stored at −70 °C until samples were shipped on dry ice to MSU for gene expression analysis. For analysis, midguts were dissected, and half of them were homogenized in Trizol reagent to extract RNA (Invitrogen, Carlsbad, CA, USA) according to the manufacturer's protocol. RNA concentration and quality (absorption ratio at 260 nm/280 nm) were spectrophotometrically measured. Equal amounts of RNA (1 μg) were added with RNAase/DNAse‐free water to yield a 12 μL mixture. To remove DNA, 2 μL of genomic DNA (gDNA) wipeout buffer was added to each mixture and incubated at 42 °C for 2 min.

Complementary DNA (cDNA) synthesis was performed with a QuantiTect Reverse Transcription Kit (Qiagen, Venlo, The Netherlands) according to the manufacturer's protocol. Briefly, 4 μL Quantiscript RT Buffer, 1 μL RT Primer Mix, and 1 μL Quantiscript Reverse Transcriptase were added to the 14 μL RNA preparation and incubated for 30 min at 42 °C for cDNA synthesis, then 3 min at 95 °C to inactivate the transcriptase, and finally stored at −80 °C. The qPCR was performed by adding 30 μL DNAase and RNase‐free water to the 20 μL cDNA and removing a 2 μL aliquot into a well. To each well, we then added a mixture containing 7.5 μL QuantiTect SYBR Green PCR Master Mix (Qiagen), 1.9 μL each of the forward and reverse primers (at 50 nm), and 6.2 μL of DNase/RNase free water. After mixing, we performed qPCR with an Applied Biosystems 7500 Real‐Time PCR System (Foster City, CA, USA). We used the following PCR conditions. After an initial phase of 50 °C for 2 min and 95 °C for 10 min, we performed 40 cycles at 95 °C for 15 s and ended at 60 °C for 60 s. Melting curves were recorded: 95 °C (15 s) to 60 °C (15 s) and back to 95 °C (15 s).

Equal numbers of bees given each treatment were run on each 96‐well plate with all six genes. Each gene per sample was run in duplicate but if the coefficient of variation percentage (CV%, standard deviation/average) was higher than 8% we then reran the sample for that gene. This happened in less than 1% of samples. We calculated gene fold differences using the 2−^∆∆*Ct*^ method of Schmittgen and Livak,^47^ using Eqn (1). Sample A consisted of our treatments (0‐40, IP‐0, or IP‐40) and sample B consisted of our control (0‐0). For each gene, based upon visual inspection of the data, we made limited *post hoc* contrast tests that we corrected for potential type I error, using the Dunn–Sidak method.^48^ We denote analyses that pass this correction as DS. We ran the full model with all interactions and used stepwise elimination of non‐significant interactions. We applied the Wald test to determine if colony (a random effect) explained a significant amount of model variance.

Bees that had their genes analyzed were separate from those that had the spores counted because we froze the abdomens in liquid nitrogen. Dissecting out their midguts to make spore counts would have likely led to RNA degradation and increased the variability in the amount of tissue being used for the qPCR analyses of gene expression. We, therefore, relied upon the major differences in spore counts, between the treatments that we used (see later).

(1) $$\begin{array}{rcl} \text{Fold change} & = & 2^{-∆∆\mathrm{Ct}}\\ & = & [\left(C_{T}\;\text{gene of interests}-C_{T}\;\text{internal control}]\right)\;\text{sample}\;A\\ & & -\;\left(C_{T}\;\text{gene of interest}-C_{T}\;\text{internal control}\right)\;\text{sample}\;B] \end{array}$$

### 2.5 Experiment 1 (E1): *in vitro* larval immune priming followed by incubator rearing

This experiment tested the efficacy of immune priming larvae in highly controlled *in vitro* rearing conditions in which there were no nurse bees to potentially remove the immune priming treatment. We used eight colonies (four for survival experiments and four for spore count analyses, Table S1). We obtained frames of brood comb and transferred first instar larvae (1‐day post egg hatching) from the comb to 24‐well cell culture plates. We followed the *in vitro* methods described in Eiri *et al*.^31^ We maintained sterile conditions and carried out all grafting, feeding, and transferring in a sterile laminar flow hood (AirClean, model 600, Creedmoor, North Carolina, USA). All equipment, including cages for holding adults, cell culture plates, glassware, and pipettes were regularly sterilized with 10% bleach solution (soaked for 30 min followed by repeated rinses with deionized water), then 70% ethanol, followed by 1‐h ultraviolet (UV) treatment and drying in the hood.

We gave larvae one of two treatments at 3 days post‐egg hatching. Control larvae were given 10 μL of sterile ddH_2_O (‘0’) with no spores dispensed onto the 100 μL of brood food (basic larval diet) immediately around the larvae.^49^ The ‘IP’ treated larvae received 40 000 heat‐killed *Nosema* spores in the 10 μL of sterile ddH_2_O dispensed onto brood food. After 24 h, we visually observed that each larva had consumed most of the liquid in its cell. We chose 40 000 spores per larva based on its potentially immune‐activating effect^31^ and because it falls within the standard range of spore doses used to infect worker bees with *N. ceranae*.^37^ Each larva was haphazardly assigned to one of the treatment groups at the beginning of each trial.

We challenged 1‐day‐old adult bees with live *Nosema* spores. We chose 1‐day‐old bees because feeding bees of this age with live *Nosema* spores has been shown to result in higher subsequent *Nosema* spore loads per bee than feeding 5‐day‐old bees with *Nosema* spores.^50^ We placed each bee inside a sterile clear plastic snap cap vial (18 mL) with a plastic lid with pierced small holes, into one of which we inserted a 100 μL pipette tip containing 2.0 μL of 50% (*w/w*) sucrose solution containing either 40 000 freshly harvested spores (see earlier) or no spores. Because newly emerged bees tend not to consume much sucrose solution, we placed these vials in a tray inside an incubator at 34 °C and 50% relative humidity (RH) until all bees had consumed the treatment (pipette tips empty). This usually took no more than 2 h. We also placed multiple empty vials (no bees) with the same treatments in pipette tips and confirmed that there was negligible evaporation of the sugar solutions during the same period.

We then placed bees into sterile plastic cages (12 cm × 8 cm × 12 cm, 25 bees per cage), with tissue paper placed on the floor to facilitate cleaning. All adults, regardless of treatment, were given 2.0 m sucrose and bee bread (consisting of 30% 2.0 m sucrose and 70% pollen *w/w*) *ad libitum*.^51^ Pollen is an important food source, particularly for young bees, and was therefore provided to bees (1.5 g of organic pollen freshly ground and placed inside a 2.5 mL centrifuge tube). This pollen came from a single batch that was commercially irradiated to kill potential pathogens such as *N. ceranae*.^31^ Each cage consisted of one treatment, and cage assignments were haphazard. Each day, we changed the paper on the cage floor to maintain cleanliness and provided new pollen, as needed. Upon death, bees were immediately placed in individually marked vials and frozen at −20 °C until they were thawed and dissected for hemocytometer spore counting (see earlier).

To measure the effects of our IP treatment on gene expression, in separate trials, we gave larvae 3 days post egg hatching either control (0) or IP treatments (IP), as described earlier. These larvae were reared on the same sterile 24‐well cell culture plates in the same incubator and handled identically by the same researcher. Upon adult emergence, we placed them in liquid nitrogen and processed them as described earlier.

### 2.6 Experiment 2 (E2): *in vivo* larval immune priming followed by incubator rearing

Experiment 2 explored the efficacy of vaccinating larvae reared in ten natural colonies. To obtain larvae of the same age, we placed an empty worker frame in the colony, removed the frame 6 days later, and visually determined that the larvae, based on size, were 3 days old. We selected brood patches with approximately 300 three‐day‐old larvae and divided them into two separate sections. A clear acetate sheet was placed over the brood patch and marked to indicate larvae locations. Each section was haphazardly assigned to one of the two treatments. With a micropipette, we placed 2 μL of sterile 50% sucrose solution containing either no spores (0) or 40 000 autoclaved spores (IP) next to each larva. A previous study showed that nurses did not remove much of such food, because at least 80% of such provided food was consumed by larvae.^52^ The comb was then returned to the colony for 5 days, after which it was moved to an incubator at 34 °C and 50% RH.^50^ Cages made of hardware cloth (6.35 mm mesh size) were placed over each treatment area and gently pushed into the comb so that newly emerged bees from the two groups did not mix.^52^

One‐day‐old adult bees (100 per trial) were challenged by being fed live *Nosema* spores and maintained inside incubated cages as described in E1. To analyze gene expression, we collected bees at the prepupal stage (17 days after egg hatching and thus 14 days after larvae were given pure sucrose of heat‐killed spores) and at 1, 7, and 14 days of adult age. Live bees were placed in liquid nitrogen as described earlier and shipped to MSU for gene expression analyses. Adult bees were recorded as ‘right‐censored’ for the survival analysis when they were removed from cages. The remaining bees were monitored daily for mortality. Dead bees were removed and frozen for hemocytometer spore counting.

### 2.7 Experiment 3 (E3): adult immune priming followed by incubator rearing

This experiment tested the efficacy of immune priming newly emerged adults that were subsequently reared in an incubator. We collected frames of capped, purple‐eye stage brood (checked by opening a few cells) and placed them in an incubator at 34 °C and 50% RH. Frames were kept in incubators for no longer than 5 days and were inspected daily for newly emerged worker bees. We used nine colonies and collected one frame per colony.

The first phase of this experiment consisted of feeding newly emerged adult bees with the pure sucrose solution or the IP treatment. Per trial, we harvested 100 bees. Bees were placed into sterile plastic vials as described in E1 and fed with 7 μL of 2.0 m sucrose solution containing either no spores (0) or the IP treatment in 100 μL pipette tips. After multiple hours, bees in E1 and E2 eventually consumed the syrup, so we devised a faster feeding method in this experiment. To facilitate faster feeding, bee‐feeding trays were modeled after Thomas Rinderer's mass‐feeding method,^53^ which takes advantage of natural bee phototaxis.^54^ We placed strips of light‐emitting diodes along the tops of each vial. This light, shining through the pipette tips, attracted bees and increased the likelihood of ingestion. Trays were placed inside an incubator as in E1. Thirty minutes after complete ingestion of treatment (based upon careful inspection of the pipette tips), we placed the bees inside cages and maintained them as in E1. As earlier, we checked for potential evaporation of the 7 μL sucrose solution in the pipette tips, but this was negligible.

The second phase, challenging bees with live spores, occurred when bees were 7 days old. To increase bee hunger, the sucrose syringes were removed from their cages for 2 h. By using artificial light and vials placed above the syringe holes,^49^ we were able to capture bees into individual sterile plastic vials where they were given 7 μL of 2.0 m sucrose solution with no spores (0) or the same volume of 2.0 m sucrose solution with 40 000 freshly harvested spores (40). We used the light strips described earlier. Thirty minutes after all bees had completely consumed the treatments, they were returned to the cages and maintained as in E1.

To measure immune gene activation, we removed live 7 and 14 days old adult bees (five per cage) and placed them in liquid nitrogen, as in E2 and shipped them to MSU for gene expression analyses. Adult bees were recorded as ‘censored’ for the survival analysis because they were removed from cages. The remaining bees were monitored daily for mortality. Dead bees were removed and frozen for hemocytometer spore counting. In this experiment, there were seven cages of 0‐0 and IP‐0 bees that had spore counts in which nine bees had spore counts. We therefore excluded bees from these cages from all analyses.

### 2.8 Experiment 4 (E4): adult immune priming followed by colony rearing

Our final experiment tested the efficacy of immune priming in full colonies in the field. As in E3, we removed combs with purple‐eyed pupae from three different colonies and placed them in nuc boxes inside an incubator (34 °C and 50% RH). Newly emerged bees (< 1 day old) were tagged with individually numbered bee tags (*N =* 99 per group) and each bee was then gently held between two fingers and individually fed via a micropipette with 2 μL of 2.0 m sucrose solution containing no spores (0) or the IP treatment. These bees were then isolated individually for 30 min to reduce food transfer and then introduced into a medium‐strength colony (about 20 000 bees).

When bees were 7 days old, they were challenged with live *Nosema* spores (IP) or received pure sucrose solution as the control. We opened up the colony, recovered all the tagged bees, and anesthetized them on ice briefly. Each worker, as it was waking from the cold, was provided with 2 μL 2.0 m sucrose solution containing no spores (0) or 40 000 freshly harvested *Nosema* spores (40). Each bee was individually isolated for 30 min in a glass vial and then returned to its original colony. Tagged bees were surveyed every 3 days until day 39 for their presence in the colony by an observer carefully checking each side of every frame three times and they were considered ‘dead’ if they were absent in all future censuses. Bees for gene expression were sampled on days 7 and 14 by freezing them in liquid nitrogen. We severed the frozen abdomens of these bees and stored each individually in 300 μL of Invitrogen RNAlater‐ICE at −80 °C. *Nosema* spore quantification was conducted with bees (*N =* 10 bees per group) that were 20 days old (12 days post‐live spore inoculation). To minimize sampling bias, spore counts were conducted at this standardized time point when *N. ceranae* spore loads are typically high. Although some heavily infected bees may have died or left the hive before this sampling point, we still observed significantly higher spore loads in the 0‐40 group compared to the IP‐40 group. Therefore, any loss of infected individuals would likely have reduced, rather than exaggerated, the observed immune priming effect on survival. The experiment was replicated with three different colonies.

### 2.9 Statistical analyses

We used JMP Pro 16.0.0 statistical software. For E1, we assessed the effect of treatment on the number of larvae that lived to emerge as adults with a chi‐square‐test. Our null hypothesis was that an equal proportion of bees would emerge as adults in both treatments (0 and IP).

To analyze the effects of treatment on adult survival, we ran Proportional Hazards survival analyses and report the log‐rank chi‐square results. When there was a significant effect of treatment, we used Hazard Ratio analysis to make all pairwise comparisons. The Hazard Ratio indicates the increased likelihood of death for treatment A as compared to treatment B and thus a Hazard Ratio_treatment A/treatment B_ = 2.0 shows that the risk of death is twice as high in treatment A as compared to treatment B.

For all experiments, we determined the effects of treatment on spore counts by using a Mixed Model (REML algorithm) with treatment as a fixed effect and colony and cage identity as random effects to account for the potential effects of colony genetic background and bees being caged together.

To analyze gene expression for all experiments, based upon residuals analyses, we log‐transformed our fold expression data and first ran a Repeated‐Measures Mixed Model (REML algorithm) with bee identity as the repeated measure (since we measured the expression of all our target genes within each bee) nested within treatment and colony as a random effect. Each age sample was collected with a different set of bees. Treatment, gene, and the treatment × gene interaction were treated as fixed effects. We treated genes as fixed effects because *Apis mellifera* up‐regulates the five Toll AMP genes, whose expression we measured, during immune defense against *N. ceranae*.^34^ For E1, we only looked at one age group. For all other experiments, we looked at multiple age groups. Therefore, if the main model showed significant differences, we next ran Mixed Models (REML algorithm, one per gene and bee age) of log‐transformed fold gene expression with colony identity as a random effect and treatment as a fixed effect. In our models, we used Tukey's honestly significant difference (HSD) tests to make corrected pairwise comparisons.

For bees that had not yet received any treatment (1‐day‐old bees in E3 and 7 days old bees in E4), we used Univariate Repeated‐Measures models with bee ID as the repeated measure.

## 3 RESULTS

### 3.1 Experiment 1 (E1): *in vitro* larval immune priming

#### 3.1.1 Survival

**Figure 1:**
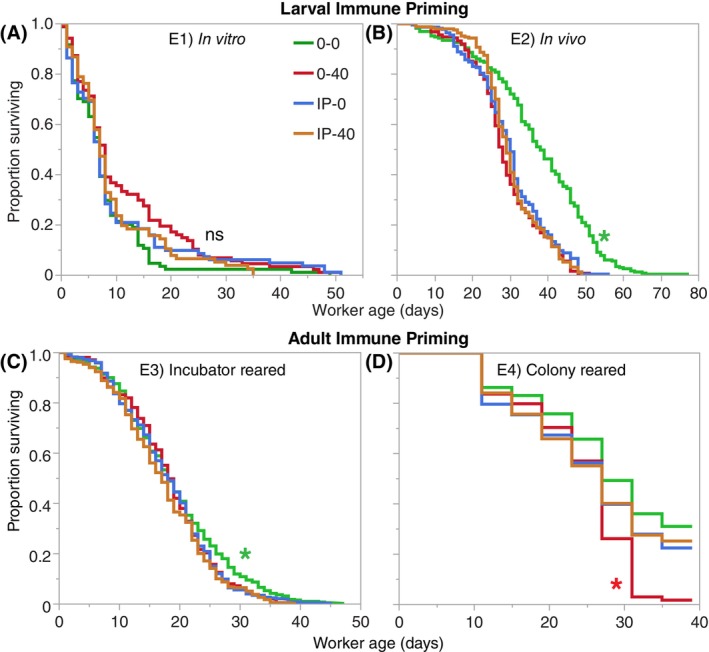
Effect of different treatments on the survival of adult bees in experiments E1–E4 (A–D). In the *in vitro* experiment, there were no significant differences between the treatments (*P* = 0.18). However, in the *in vivo* and incubator‐reared experiments, the control group (0‐0) had significantly higher survival than all other treatments, as indicated by the green stars in both experiments (E2: *P* < 0.0001; E3: *P* = 0.0004). In the colony‐reared experiment, the 0‐40 group had significantly lower survival than all other groups (*P* < 0.0001) as indicated by the red star, but the survival of the other three treatments did not differ. Analyses were conducted separately for each experiment. Sample sizes are shown in Supporting Information Table S1.

There was no significant difference between the survival of control bees fed pure sucrose (81%) or immune primed (72%) larvae to adulthood (chi‐square = 1.86, *P* = 0.17). These survival rates were similar to the 85% survival of larvae to adulthood when reared by nurse bees inside honey bee colonies.^49^, ^55^ The 51‐day maximum longevity of our adults is similar to that of naturally reared adults subsequently kept in cages.^56^ Still, our median age of death, 7–8 days for all treatment groups, was low, suggesting an effect of *in vitro* rearing. Treatment did not significantly affect adult survival (log‐rank chi‐square = 4.94, 3 df, *P* = 0.18, Fig. 1(A)).

#### 3.1.2 Infection

**Figure 2:**
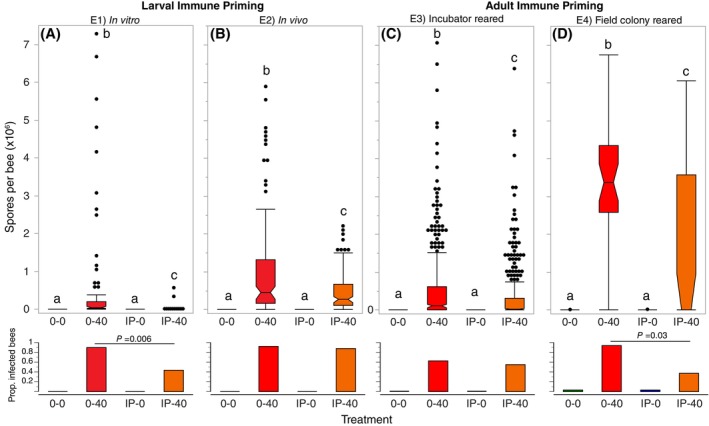
The effect of different treatments on the mean infection levels of adult bees upon death (spores per bee, outlier box plots) and on the proportion of infected bees in the four different experiments (E1–E4, A–D). A bee is defined as infected if any spores were found. Analyses were conducted separately for each of the four experiments. Different letters indicate significant differences (Tukey's HSD tests, *P* < 0.05). For the proportion of infected bees, we show the results of significant (*P* < 0.05) two‐tailed Fisher's exact tests comparing the numbers of bees in the 0‐40 and IP‐40 treatment groups. Sample sizes are shown in Supporting Information Table S1. Mean spore counts and standard errors are shown in Table S3.

There was a significant effect of treatment (*F*_3,295_ = 11.17, *P* < 0.0001) such that bees in IP‐40, IP‐0, and 0‐0 groups had significantly lower spore counts than the positive control group (0‐40), which had the highest spore counts (Tukey's HSD test, *P* < 0.05, Fig. 2(A)). On average the 0‐40 bees had 33.2 times more spores than the IP‐40 group. The IP‐40 group had a significantly lower proportion of infected bees (43%) than the 0‐40 group (90%, Fisher's two‐tailed exact test, *P* = 0.006). No bees in the IP‐0 or 0‐0 groups were infected.

#### 3.1.3 Immune gene expression

**Figure 3:**
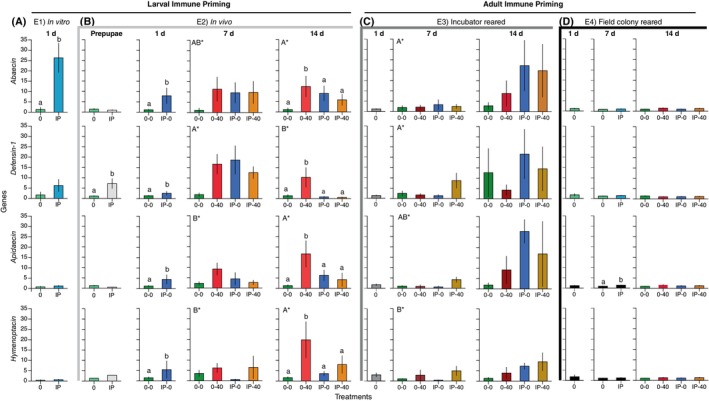
Effect of treatments on four genes in the Toll pathway that are associated with *Nosema ceranae* infection (*abaecin*, *defensin‐1*, *apidaecin*, and *hymenoptacin*) in each of the four experiments (E1–E4, A–D) at different ages (days after adult emergence), when these were examined. In every experiment, each time point compares gene expression levels at that time point with the expression of control genes (*actin* and *GAPDH*). Analyses were run separately for each experiment and different letters indicate significant differences within each experiment and time point (Tukey's HSD, *P* < 0.05). Lowercase letters show significant differences between treatments for each gene. Uppercase letters with ‘*’ show differences between genes (E2). No letters are shown if there were no significant differences. Plots show means and standard error bars. For E3 and E4, the 1‐day time point provides baseline gene expression data because these bees were collected before they received any treatment. Sample sizes are shown in Supporting Information Table S1. Means and standard errors of the fold expression levels are shown in Table S4.

In the 1‐day‐old adult bees, there was a significant effect of treatment (*F*_1,11_ = 14.81, *P* = 0.003), gene (*F*_3,35_ = 9.89, *P* < 0.0001), and the interaction treatment × gene (*F*_3,35_ = 3.36, *P* = 0.03, Fig. 3(A)) on the immune gene expression levels. Specifically, the relative levels of *abaecin* were elevated in IP bees as compared to controls (‘0’ bees, Tukey's HSD test, *P* < 0.05). None of the other focal genes showed significant expression differences between treatments (Tukey's HSD test, *P* > 0.05).

### 3.2 Experiment 2 (E2): *in vivo* larval immune priming

#### 3.2.1 Survival

There was a significant overall effect of treatment on survival (log‐rank chi‐square = 132.39, 3 df, *P* < 0.0001 DS), with the negative control group (0‐0) having significantly higher survival (median survival time of 38 days) than any other treatment group (Hazard Ratio_0‐0 compared to the other treatments_ ≤ 0.37, *P* < 0.0001, Fig. 1(B)). There was no significant difference in survival among the 0‐40, IP‐40, or IP‐0 treatment groups (Hazard Ratio_between 0‐40, IP‐40, and IP‐0_ ≥ 0.86, *P* ≥ 0.09).

#### 3.2.2 Infection

There was a significant effect of treatment (*F*_3,13_ = 47.03, *P* < 0.0001) because immune‐primed bees subsequently fed live spores (IP‐40) had significantly fewer spores than the positive control group (0‐40), which had the highest spore counts (Tukey's HSD test, *P* < 0.05, Fig. 2(B)). On average, the 0‐40 bees had two times more spores than the IP‐40 bees. The proportion of infected bees did not differ between the 0‐40 (95%) and IP‐40 (88%) groups (Fisher exact test, *P* = 0.85). The negative control groups were uninfected.

#### 3.2.3 Immune gene expression

For prepupae, there was a significant interaction of treatment × gene (*F*_3,86_ = 7.33, *P* = 0.0002) and gene (*F*_3,86_ = 7.44, *P* = 0.0002), but no significant effect of treatment (*F*_1,86_ = 0.002, *P* = 0.97) because *defensin‐1* was higher in the IP treatment than in the 0 treatment. No other genes had significantly different expression levels among treatments. By 1 day of adult age, IP‐0 bees had higher levels of expression for all four genes than 0‐0 bees (treatment effect: *F*_1,33_ = 37.52, *P* < 0.0001). However, there were no significant effects of gene (*F*_3,85_ = 0.66, *P* = 0.58) or the interaction treatment × gene (*F*_3,82_ = 1.70, *P* = 0.17). At 7‐days of adult age, there was no significant effect of treatment (*F*_3,5_ = 1.43, *P* = 0.34), but gene was significant (*F*_3,171_ = 6.49, *P* = 0.0003) because *defensin‐1* was more highly expressed than *apidaecin* or *hymenoptacin* (Tukey's HSD test, *P* < 0.05) across all treatments. The interaction of treatment × gene was not significant (*F*_9,161_ = 1.82, *P* = 0.07, Fig. 3(B)). Finally, for 14‐day‐old adults, there was a significant effect of gene (*F*_3,175_ = 7.33, *P* = 0.0001) because *defensin‐1* expression was now significantly lower than expression of all other genes over all treatments (Tukey's HSD test, *P* < 0.05). Treatment was also significant because 0‐40 bees had overall higher levels of expression of the four Toll genes than any other treatment. For this age group, there was no significant interaction of treatment × gene (*F*_9,166_ = 1.01, *P* = 0.43).

### 3.3 Experiment 3 (E3): adult immune priming followed by incubator rearing

#### 3.3.1 Survival

For bees that were immune primed as adult, there was a significant effect of treatment on bee survival (log‐rank chi‐square = 18.02, 3 df, *P* = 0.0004) because control bees (0‐0) lived significantly longer than all other groups (Hazard Ratio_0‐0 compared to the other treatments_ ≤ 0.84, *P* ≤ 0.02, Fig. 1(C)).

#### 3.3.2 Infection

There were significant differences in infection levels between treatments (*F*_3,1433_ = 61.23, *P* < 0.0001), with 0‐40 bees having significantly more (1.5 times) spores than IP‐40 bees (Tukey's HSD, *P* < 0.05, Fig. 2(C)). IP‐0 and 0‐0 bees were not infected. There was no difference in the number of 0‐40 and IP‐40 bees that were infected (Fisher's two‐tailed exact test, *P* = 0.81). There was no significant difference in the proportion of IP‐40 bees (55%) and 0‐40 bees (63%) that were infected (Fisher's two‐tailed exact test, *P* = 0.03).

#### 3.3.3 Immune gene expression

For 1‐day‐old adults, there were no significant differences in the expression of the four Toll genes (*F*_3,54_ = 0.005, *P* = 1.0). At 7 days of age, there were significant differences in the expression of the Toll genes (*F*_3,64_ = 3.72, *P* = 0.02) because *defensin‐1* and *abaecin* had higher expression levels than *hymenoptacin* (Fig. 3(C)). For these 7‐day‐old bees, there was no significant effect of treatment (*F*_3,8_ = 1.53, *P* = 0.28) or the interaction of treatment × gene (*F*_9,54_ = 1.03, *P* = 0.43). By the age of 14 days, there were no significant effects of treatment (*F*_3,11_ = 1.71, *P* = 0.22), gene (*F*_3,40_ = 1.38, *P* = 0.26), or the interaction of treatment × gene (*F*_9,36_ = 0.20, *P* = 0.99).

### 3.4 Experiment 4 (E4): adult immune priming followed by colony rearing

#### 3.4.1 Survival

There was a significant effect of treatment on bee survival (log‐rank chi‐square = 33.19, 3 df, *P* < 0.0001 DS) because the positive control (0‐40) bees had significantly lower survival than 0‐0, IP‐0, and IP‐40 bees (Hazard Ratio_0‐40/0‐0_ = 1.74, *P* < 0.0001; Hazard Ratio_0‐40/IP‐0_ = 1.47, *P* < 0.0001; and Hazard Ratio_0‐40/IP‐40_ = 1.50, *P* < 0.0001, Fig. 1(D)).

#### 3.4.2 Infection

There was a significant effect of treatment (*F*_3,126_ = 46.73, *P* < 0.0001) with 0‐40 bees having significantly higher infection levels than IP‐40 bees (Tukey's HSD test, *P* > 0.05). There was no significant difference between IP‐0 and 0‐0 bees, which had almost no infections (Tukey's HSD test, *P* > 0.05, Fig. 2(D)). On average, the 0‐40 bees had 2.3 times more spores than the IP‐40 bees. A significantly lower proportion of IP‐40 bees (37%) were infected compared to the 0‐40 bees (94%, Fisher's two‐tailed exact test, *P* = 0.03). The negative control groups had very low levels of infection: 3.1% of 0‐0 bees and 3.1% of IP‐0 bees.

#### 3.4.3 Immune gene expression

Before they received any treatment, there was no significant difference in the expression of our target immune genes (1‐day‐old bees, *F*_3,51_ = 0, *P* = 1.0, Fig. 3(D)). The 7‐day‐old adult bees had been immune primed but had not yet received any live spores. In these 7‐day‐old adults, there was a significant effect of treatment (*F*_1,68_ = 4.60, *P* = 0.036) but no significant effect of gene (*F*_3,213_ = 2.13, *P* = 0.10) or the interaction of treatment × gene (*F*_3,210_ = 2.17, *P* = 0.09) on immune gene expression. Specifically, IP bees had significantly higher levels of *apidaecin* than control (0) bees (Tukey's HSD test, *P* < 0.05). In the 14‐day‐old adults, there were no significant effects of treatment (*F*_3,66_ = 0.49, *P* = 0.69), gene (*F*_3,213_ = 0.17, *P* = 0.92), or the interaction of treatment × gene (*F*_9,204_ = 1.29, *P* = 0.24) on immune gene expression levels.

## 4 DISCUSSION

Our results show that honey bee workers primed with autoclaved *N. ceranae* spores and then challenged with live‐spores had significantly lower infection levels than bees that did not receive this initial priming. This finding supports the general principle of immune priming in insects, whereby prior exposure to sublethal pathogen material can enhance subsequent disease resistance. Notably, the developmental stage at which priming was administered appeared to influence its effectiveness. The strongest protection occurred in larvae reared entirely in the laboratory, which showed a 97% reduction in mean spore counts, with spore counts 33.2‐fold higher in unprimed bees fed live spores (positive controls). This was followed by newly emerged adults placed in field colonies (56% reduction, 2.3‐fold higher in positive controls), larvae fed within field colonies (52% reduction, 2.1‐fold higher in positive controls), and newly emerged adults reared in incubated laboratory cages (34% reduction, 1.5‐fold higher in positive controls). The reduced longevity observed in *in vitro*‐reared bees (E1) compared to other experiments likely reflects physiological stress associated with artificial rearing as compared to natural rearing in a honey bee colony.^57^ Thus, one might expect *in vitro*‐reared bees to have weaker immune systems and therefore benefit less from immune priming. Nonetheless, they had the strongest response to immune priming, with a 97% reduction in spore loads as compared to the positive controls. Larval immune priming therefore often provided greater protection than adult immune priming, especially when performed in a controlled, *in vitro* setting, where nurse bees could not remove or dilute the priming material. Although we do not yet know whether this effect is specific to *N. ceranae* or might extend to other pathogens, every experiment in this study revealed a clear protective benefit in infection levels.

We observed high variation in infection levels, as found in other studies.^25^, ^58^ Measuring spore counts at death likely contributed to this variation since *Nosema* infection increases with age. Yet there were no significant survival differences among treatments, apart from the mostly uninfected control (0‐0). Thus, the three spore‐fed treatments (IP‐0, IP‐40, and 0‐40) had similar age distributions when spore loads were assessed, indicating that bees from each treatment group were sampled at comparable ages. Our findings demonstrate that immune priming significantly reduces spore counts, whether delivered *in vitro* to larvae or to newly emerged adults in the field.

Despite variation in colony replication across experiments, our results are supported by effect size and power analyses based on biologically independent replicates at the colony level. Each colony contributed separate groups of bees to each treatment, enabling paired comparisons of mean responses within colonies. For spore load, the paired effect sizes (Cohen's *d*_z_)^59^ for the contrast between challenged, unprimed (0‐40) and challenged, immune‐primed (IP‐40) treatments ranged from 0.91 to 7.45 across E1–E4, with corresponding statistical power estimates from 30% to > 99% Notably, in E4, where only three colonies were available due to field constraints, the effect size was exceptionally large (*d*_z_ = 7.45), yielding > 99% power despite the low replication.

In contrast, the effect sizes observed in survival experiments were generally smaller (−0.37 to 1.32), and power was correspondingly lower (7% to 66%). This variation in power is consistent with both the magnitude of observed effects and the inherent differences between laboratory and field conditions.

### 4.1 Gene expression

Our results partly align with previous findings that *N. ceranae* infection elevates AMP genes,^44^ although no single AMP in our study emerged as a reliable marker of immune priming. Across various developmental stages and rearing conditions, priming sometimes increased the expression of *abaecin*, *apidaecin*, *defensin‐1*, and *hymenoptacin*; however, these effects depended on the timing of immune priming and the age at sampling. These findings align with other studies showing that different AMPs can be variably induced depending on infection dynamics and the timing of infection.^34^ In our experiments, 1‐day‐old adults from *in vitro* and *in vivo* primed larvae showed consistently higher *abaecin* expression than controls. However, this elevated *abaecin* expression was not detected at subsequent time points in the *in vivo* experiment, which may account for the lack of elevated *abaecin* expression in 7‐day‐old and 14‐day‐old bees in the adult immune priming experiments (Fig. 3(A),(B)). Because adult‐primed bees were fed heat‐killed spores as 1‐day‐old adults, *abaecin* expression levels for immune‐primed bees were not measured on the same day (Fig. 3(C),(D)).

In the *in vivo* experiment, *defensin‐1* was strongly expressed in immune‐primed bees when they were prepupae and 1‐day‐old adults, but this elevation did not persist at 7 or 14 days (Fig. 3(B)). However, the positive control bees that were not immune primed but were fed live spores were infected by day 14, and had elevated *defensin‐1* expression (Fig. 3(B)). These results match those of Hinshaw *et al*.^60^ who reported that *defensin‐1* expression in honey bees increased with higher levels of *N. ceranae* infection. Because *defensin‐1* expression increases in gut epithelia following contact with trypanosome cyst walls,^34^ we speculate that contact with the heat‐killed *Nosema* spore walls may have led to increased defensin‐1 expression. Relevantly, in the mealworm beetle, *tenecin 1* (a member of the *defensin‐1* family) facilitates transgenerational immune priming.^61^ A similar elevation of *apidaecin* occurred for *in vivo* experiment 1‐day‐old bees that were immune primed as larvae and at day 14 for the positive control bees (Fig. 3(B)). In the adult immune priming experiments, there were no significant Toll gene expression differences in the incubator reared bees (E3), and only a slight elevation of *apidaecin* in immune‐primed bees at 7‐day‐old adults (Fig. 3(C),(D)).

Thus, no strong, consistent differences emerged for the four genes we examined across all experiments. Future work employing transcriptomic profiling and protein‐level analyses could help to clarify the role of *defensin‐1* and *apidaecin* in sustaining protection against *N. ceranae*. Immune priming in honey bees can change over time and be context‐dependent,^34^ and the role of Toll genes in *N. ceranae* infections is not fully elucidated. Our findings also suggest only an association between certain Toll genes and immune priming and we did not measure Spätzle proteolytic activation, which is essential for initiating Toll signaling and contributes to immune responses in *Apis mellifera*.^62^

Beyond AMP expression, immune priming in honey bees likely involves broader innate immune adaptations, including mechanisms analogous to trained immunity or immune memory. In invertebrates, such responses can involve epigenetic modifications, regulation by microRNAs, altered hemocyte activity, and sustained activation of pattern recognition receptors.^7^ The protection we observed following priming suggests that these memory‐like processes may contribute to enhanced resistance, warranting further investigation.

### 4.2 Survival: costs and trade‐offs

An important aspect of immune priming research involves understanding potential costs, which can manifest as reduced survival or reproductive output. Several insect species exhibit such trade‐offs; for example, red flour beetles that received transgenerational immune priming produced fewer first‐generation offspring.^63^ However, the offspring of bumble bee queens primed against a bacteria, *Arthrobacter globiformis,* did not show survival costs, but had decreased resistance to a parasite, *Crithidia bombi*.^30^ In the ant *Crematogaster scutellaris*, immune‐primed queens produced more resilient workers but did not suffer reduced offspring production.^64^ These examples indicate that immune priming outcomes can be context‐ and species‐dependent.

In honey bees, our findings suggest that immune priming can sometimes incur survival costs. Under *in vitro* conditions (E1), survival did not differ significantly among treatments, but in cage and field experiments, unchallenged control bees generally outlived spore‐fed or immune‐primed bees. Factors such as propolis use, honey consumption, or colony‐level behaviors may mitigate these costs in natural settings.^65^, ^66^ Moreover, *Nosema* infections do not always reduce longevity, and differences can arise between cage and colony conditions.^67^ Further work is needed to clarify how honey bees might balance these immunological benefits against potential survival trade‐offs.

Finally, our findings offer promising applications for improving honey bee health through targeted immune priming. The protective effects of oral exposure to heat‐killed *Nosema* spores – particularly when administered early in development – support the potential for feed‐based prophylactic strategies. Incorporating wimmune elicitors into larval or young adult diets during periods of peak brood‐rearing or heightened infection risk, such as in the spring,^68^, ^69^ could enhance colony resilience if the benefits of reduced infection levels outweigh the survival costs. The field colony experiment (E4) provides the most realistic guidance and, in this experiment, there was no increased mortality due to immune priming alone. Because implementing such strategies requires large scale production of inactivated *Nosema*, transgenerational immune priming of honey bee queens may ultimately be more practical. Future work should therefore focus on optimizing delivery protocols, testing colony‐level outcomes, and determining whether maternal immune priming can confer long‐term protection to offspring across developmental stages and seasons.

## CONFLICT OF INTEREST

The authors declare no competing interests.

## Supporting information


**Table S1.** Sample sizes for all four experiments. In all experiments, the bees used were approximately equally distributed among the colonies used. For the gene expression, analyses, we used workers from a randomly selected subset of the colonies used in each experiment.
**Table S2.** Primers used to measure immune gene activation (AMP = antimicrobial peptide).
**Table S3.** Mean spore counts of bees in all treatments in all experiments.
**Table S4.** Results of immune gene expression analyses from all experiments. The mean ± 1 standard error fold change values are shown. For ease of comparison, similar columns are aligned. For experiment 1, bees were collected within 24 h of emergence to measure immune gene expression and therefore corresponded to 1‐day‐old adults.
