# Supplementary material for: Immune priming of honey bees protects against a major microsporidian pathogen
Source: Pest Manag Sci. 2025 Aug 4;81(12):7939–49. doi: 10.1002/ps.70106 (PMC12618913; doi:10.1002/ps.70106)
Supplement: Supplementary file 1 — Table S1. Sample sizes for all four experiments. In all experiments, the bees used were approximately equally distributed among the colonies used. For the gene expression, analyses, we used workers from a randomly selected subset of the colonies used in each experiment. Table S2. Primers used to measure immune gene activation (AMP = antimicrobial peptide). Table S3. Mean spore counts of bees in all treatments in all experiments. Table S4. Results of immune gene expression analyses from all experiments. The mean ± 1 standard error fold change values are shown. For ease of comparison, similar columns are aligned. For experiment 1, bees were collected within 24 h of emergence to measure immune gene expression and therefore corresponded to 1‐day‐old adults. [file PS-81-7939-s001.docx]

**SUPPLEMENTAL INFORMATION**

**Table S1**. Sample sizes for all four experiments. In all experiments, the bees used were approximately equally distributed among the colonies used. For the gene expression, analyses, we used workers from a randomly selected subset of the colonies used in each experiment.

| **Survival analysis** | | | | | |
| --- | --- | --- | --- | --- | --- |
| Experiment | Treatment | No. of bees in survival analyses | No. of colonies in survival analyses & (no. of trials) | No. of bees in spore count analyses | No. of colonies in spore count analyses & (no. of trials) |
| E1 | 0-0 | 84 | 4 (5) | 84 | 4 (5) |
|  | 0-40 | 87 |  | 87 |  |
|  | IP-0 | 81 |  | 81 |  |
|  | IP-40 | 76 |  | 76 |  |
|  |  |  |  |  |  |
| E2 | 0-0 | 229 | 10 (10) | 123 | 5 (5) |
|  | 0-40 | 249 |  | 125 |  |
|  | IP-0 | 224 |  | 121 |  |
|  | IP-40 | 247 |  | 113 |  |
|  |  |  |  |  |  |
| E3 | 0-0 | 345 | 9 (10) | 345 | 9 (18) |
|  | 0-40 | 416 |  | 416 |  |
|  | IP-0 | 313 |  | 313 |  |
|  | IP-40 | 368 |  | 368 |  |
|  |  |  |  |  |  |
| E4 | 0-0 | 219 | 3 (3) | 32 | 3 (3) |
|  | 0-40 | 233 |  | 32 |  |
|  | IP-0 | 236 |  | 32 |  |
|  | IP-40 | 214 |  | 32 |  |
| **Gene expression analysis** | | | | | |
| Experiment | Treatment | No. of bees in gene analyses | No. of colonies in gene analyses & (no. of trials) |  |  |
| E1 | 0 | 7 | 4 (4) |  |  |
|  | IP | 9 |  |  |  |
|  |  |  |  |  |  |
| E2 | 0-0 | 44 | 3 (3) |  |  |
|  | 0-40 | 24 |  |  |  |
|  | IP-0 | 47 |  |  |  |
|  | IP-40 | 24 |  |  |  |
|  |  |  |  |  |  |
| E3 | 0-0 | 29 | 7 (7) |  |  |
|  | 0-40 | 12 |  |  |  |
|  | IP-0 | 11 |  |  |  |
|  | IP-40 | 12 |  |  |  |
|  |  |  |  |  |  |
| E4 | 0 | 54 | 3 (3) |  |  |
|  | IP | 36 |  |  |  |
|  | 0-0 | 18 |  |  |  |
|  | 0-40 | 18 |  |  |  |
|  | IP-0 | 18 |  |  |  |
|  | IP-40 | 18 |  |  |  |

**Table S2.** Primers used to measure immune gene activation (AMP=antimicrobial peptide).

| **Gene type** | **Name** | **Direction** | **No. of bases** | **Sequence** | **Reference** |
| --- | --- | --- | --- | --- | --- |
| AMP | *Abaecin*-F | Forward | 20 | CAG CAT TCG CAT ACG TAC CA | Evans et al. (2006) |
| AMP | *Abaecin*-R | Reverse | 20 | GAC CAG GAA ACG TTG GAA AC |  |
| AMP | *ApidNT*-F | Forward | 23 | TTT TGC CTT AGC AAT TCT TGT TG | Simone et al. (2009) |
| AMP | *ApidNT*-R | Reverse | 21 | GTA GGT CGA GTA GGC GGA TCT |  |
| AMP | *Defensin-1*-F | Forward | 20 | TGC GCT GCT AAC TGT CTC AG | Evans et al. (2006) |
| AMP | *Defensin-1*-R | Reverse | 20 | AAT GGC ACT TAA CCG AAA CG |  |
| AMP | *Hymenoptacin*-F | Forward | 20 | CTC TTC TGT GCC GTT GCA TA | Evans et al. (2006) |
| AMP | *Hymenoptacin*-R | Reverse | 20 | GCG TCT CCT GTC ATT CCA TT |  |
| Control | *GAPDH*-F | Forward | 20 | GAT GCA CCC ATG TTT GTT TG | Scharlaken et al. (2008) |
| Control | *GAPDH*-R | Reverse | 20 | TTT GCA GAA GGT GCA TCA AC |  |
| Control | *Actin*-F | Forward | 22 | TTG TAT GCC AAC ACT GTC CTT T | Simone et al. (2009) |
| Control | *Actin*-R | Reverse | 20 | TGG CGC GAT GAT CTT AAT TT |  |

**Table S3.** Mean spore counts of bees in all treatments in all experiments.

| **Experiment** | **Treatment** | **No. of bees** | **Mean spore count per bee**  **(mean±1 standard error)** |
| --- | --- | --- | --- |
| **1** | 0-0 | 84 | 0±0 |
| ***In vitro*** | 0-40 | 87 | 544,195±151,989 |
|  | IP-0 | 81 | 0±0 |
|  | IP-40 | 76 | 16,382±8,727 |
|  |  |  |  |
| **2** | 0-0 | 123 | 0±0 |
| ***In vivo*** | 0-40 | 125 | 992,320±115,514 |
|  | IP-0 | 121 | 0±0 |
|  | IP-40 | 113 | 479,425±49,106 |
|  |  |  |  |
| **3** | 0-0 | 417 | 516±322 |
| **Incubator reared** | 0-40 | 416 | 520,438±46,792 |
|  | IP-0 | 403 | 1,335±715 |
|  | IP-40 | 368 | 342,673±38,894 |
|  |  |  |  |
| **4** | 0-0 | 32 | 547* |
| **Field colony reared** | 0-40 | 32 | 3,401,563±252,030 |
|  | IP-0 | 32 | 703* |
|  | IP-40 | 35 | 1,482,643±362,562 |

* Spores only found in 1 bee, insufficient to calculate standard error.

**Table S4**. Results of immune gene expression analyses from all experiments. The mean±1 standard error fold change values are shown. For ease of comparison, similar columns are aligned. For experiment 1, bees were collected within 24 h of emergence to measure immune gene expression and therefore corresponded to 1-day-old adults.

| **Experiment** | **Gene** | **No. of bees** |  | **0, 1 day adult** |  |  |  |  |  | **IP, 1 day adult** |  |  |  |  |
| --- | --- | --- | --- | --- | --- | --- | --- | --- | --- | --- | --- | --- | --- | --- |
| **1** | *abaecin* | 12 |  | 1.34±1.03 |  |  |  |  |  | 26.35±7.09 |  |  |  |  |
| ***In vitro*** | *apidaecin* | 15 |  | 0.79±0.28 |  |  |  |  |  | 1.23±0.31 |  |  |  |  |
|  | *defensin* | 16 |  | 1.70±1.43 |  |  |  |  |  | 6.26±2.98 |  |  |  |  |
|  | *hymenoptacin* | 14 |  | 0.38±0.27 |  |  |  |  |  | 0.65±0.24 |  |  |  |  |
|  |  |  |  |  |  |  |  |  |  |  |  |  |  |  |
| **2** | **Gene** | **No. of bees** | **0-0, prepupae** | **0-0, 1 day adult** | **0-0, 7 day adult** | **0-0, 14 day adult** | **0-40, 7 day adult** | **0-40, 14 day adult** | **IP-0, prepupae** | **IP-0, 1 day adult** | **IP-0, 7 day adult** | **IP-0, 14 day adult** | **IP-40, 7 day adult** | **IP-40, 14 day adult** |
| ***In vivo*** | *abaecin* | 139 | 1.41±0.31 | 1.19±0.31 | 1.09±0.18 | 1.14±0.25 | 11.31±5.84 | 12.3±5.03 | 0.96±0.26 | 7.96±3.77 | 9.57±4.96 | 8.95±3.65 | 9.74±5.38 | 5.83±2.68 |
|  | *apidaecin* | 139 | 1.25±0.23 | 1.27±0.36 | 2.25±0.81 | 1.33±0.44 | 9.17±2.94 | 16.67±6.39 | 0.48±0.09 | 4.47±2.24 | 4.44±3.04 | 6.3±2.57 | 2.68±1.13 | 4.21±3.24 |
|  | *defensin* | 137 | 1.2±0.19 | 1.39±0.24 | 1.94±0.81 | 1.38±0.56 | 16.7±4.79 | 10.29±4.72 | 7.21±2.41 | 2.58±0.91 | 18.68±6.88 | 0.83±0.3 | 12.57±2.87 | 0.6±0.22 |
|  | *hymenoptacin* | 139 | 1.37±0.24 | 1.51±0.45 | 3.57±1.54 | 1.66±0.44 | 6.21±2.25 | 19.96±8.77 | 2.84±1.45 | 5.43±4.24 | 0.58±0.23 | 3.62±1.12 | 6.47±5.5 | 8.01±4.3 |
|  |  |  |  |  |  |  |  |  |  |  |  |  |  |  |
| **3** | ***Gene*** | **N Rows** | **—** | **0, 1 day adult** | **0-0, 7 day adult** | **0-0, 14 day adult** | **0-40, 7 day adult** | **0-40, 14 day adult** | **—** | **—** | **IP-0, 7 day adult** | **IP-0, 14 day adult** | **IP-40, 7 day adult** | **IP-40, 14 day adult** |
| **Incubator reared** | *abaecin* | 63 | **—** | 1.21±0.17 | 2.13±1.09 | 3.17±1.9 | 2.11±0.95 | 8.79±6.17 | **—** | **—** | 3.28±2.41 | 22.27±12.22 | 2.42±1.04 | 19.82±12.79 |
|  | *apidaecin* | 55 | **—** | 1.7±0.38 | 1.04±0.31 | 2.06±1.35 | 0.9±0.7 | 9.19±6.6 | **—** | **—** | 0.79±0.28 | 27.82±0.57 | 4.16±1.27 | 16.94±15.72 |
|  | *defensin* | 62 | **—** | 1.29±0.23 | 2.8±1.33 | 18.51±17.47 | 1.74±0.65 | 4.25±2.55 | **—** | **—** | 1.38±0.54 | 21.65±11.96 | 8.65±3.63 | 14.52±10.63 |
|  | *hymenoptacin* | 54 | **—** | 2.95±0.96 | 1.08±0.24 | 1.45±0.64 | 2.83±2.5 | 3.93±2.8 | **—** | **—** | 0.41±0.14 | 7.26±1.49 | 4.89±2.29 | 9.37±4.34 |
|  |  |  |  |  |  |  |  |  |  |  |  |  |  |  |
| **4** | ***Gene*** | **N Rows** | **—** | **0, 1 day adult** | **0, 7 day adult** | **0-0, 14 day adult** | **—** | **0-40, 14 day adult** | **—** | **—** | **IP, 7 day adult** | **IP-0, 14 day adult** | **—** | **IP-40, 14 day adult** |
| **Field colony reared** | *abaecin* | 162 | **—** | 1.2±0.19 | 0.94±0.06 | 0.99±0.09 | **—** | 1.5±0.21 | **—** | **—** | 1.13±0.11 | 0.99±0.13 | **—** | 1.38±0.14 |
|  | *apidaecin* | 162 | **—** | 1.2±0.17 | 0.92±0.09 | 0.99±0.08 | **—** | 1.63±0.25 | **—** | **—** | 1.39±0.13 | 1.2±0.2 | **—** | 1.28±0.19 |
|  | *defensin* | 162 | **—** | 1.71±0.56 | 1.22±0.1 | 1.47±0.16 | **—** | 1.08±0.12 | **—** | **—** | 1.45±0.13 | 1.13±0.14 | **—** | 1.21±0.17 |
|  | *hymenoptacin* | 162 | **—** | 1.81±0.88 | 1.14±0.09 | 1.07±0.09 | **—** | 1.32±0.22 | **—** | **—** | 1.26±0.21 | 1.16±0.12 | **—** | 1.37±0.22 |
